# Diagnostic Accuracy of a Novel Point of Care High‐Sensitivity Troponin Assay in the Prehospital Environment

**DOI:** 10.1111/acem.70213

**Published:** 2026-01-13

**Authors:** John Gilman, Abdulrhman Alghamdi, Mark Hann, Edward Carlton, Jamie G. Cooper, Eloïse Cook, Aloysius Niroshan Siriwardena, John Phillips, Alexander Thompson, Steve Bell, Kim Kirby, Andy Rosser, Richard Body

**Affiliations:** ^1^ Department of Laboratory Medicine Northern Care Alliance NHS Foundation Trust Salford UK; ^2^ College of Applied Medical Sciences, King Saud Bin Abdulaziz University for Health Sciences Riyadh Saudi Arabia; ^3^ King Abdullah International Medical Research Center Riyadh Saudi Arabia; ^4^ Division of Population Health Health Services Research & Primary Care, the University of Manchester Manchester UK; ^5^ University of Bristol Medical School, Translational Health Sciences, Southmead Hospital Learning and Research Bristol UK; ^6^ Emergency Department Aberdeen Royal Infirmary, NHS Grampian Aberdeen UK; ^7^ School of Medicine, Medical Sciences and Nutrition University of Aberdeen Aberdeen UK; ^8^ Emergency Department Manchester University NHS Foundation Trust Manchester UK; ^9^ Community and Health Research Unit, School of Health and Social Care, University of Lincoln Lincoln UK; ^10^ The Ticker Club (A Cardiac Patient Support Group), Wythenshawe Hospital Manchester UK; ^11^ Medical Directorate, North West Ambulance Service NHS Foundation Trust Bolton UK; ^12^ Centre for Health and Clinical Research, School of Health and Social Wellbeing, University of the West of England Bristol UK; ^13^ West Midlands Ambulance Service University NHS Foundation Trust Brierley Hill UK; ^14^ Division of Cardiovascular Science The University of Manchester Manchester UK

**Keywords:** acute coronary syndromes, acute myocardial infarction, diagnosis, emergency medical services, high sensitivity troponins, prehospital care, sensitivity and specificity, troponins

## Abstract

**Objective:**

To evaluate the diagnostic accuracy of a novel point of care (POC) high‐sensitivity troponin (hs‐cTn) assay, used alone or incorporated within validated decision aids, for acute myocardial infarction (AMI) in the prehospital setting.

**Methods:**

A pre‐specified secondary analysis of the Prehospital Evaluation of Sensitive Troponin (PRESTO) prospective diagnostic accuracy study, conducted in four ambulance services and 12 Emergency Departments (EDs; February 2019–March 2020). Paramedics included consenting adults with suspected AMI and no other reason for conveyance. Clinical data and venous blood were collected at the scene, and samples conveyed to hospital with participants. Plasma samples were later analyzed for hs‐cTn using a novel POC hs‐cTn assay (Abbott Point of Care i‐STAT hs‐TnI). The target condition was an adjudicated index diagnosis of type 1 AMI.

**Results:**

Of 817 consenting participants, 704 were eligible for inclusion in this analysis, with type 1 AMI occurring in 89 (12.6%). At the limit of detection (< 2 ng/L), POC hs‐cTn had 100.0% sensitivity (95% CI 95 9%–100.0%) but only 4.6% specificity (95% CI 3.1%–6.5%). A Troponin‐only Manchester Acute Coronary Syndromes (T‐MACS) very‐low risk outcome identified 134 (19.7%) patients for non‐conveyance with 98.9% sensitivity (95% CI 94.9%–100.0%), 99.3% negative predictive value (NPV, 95% CI 95.0%–99.9%), and 22.5% specificity (95% CI 19.2%–26.1%). A low‐risk modified HEART score identified 150 (22.0%) patients with 93.2% sensitivity (95% CI 85.8%–97.5%), 96.0% NPV (91.6%–98.1%), and 24.3% specificity (95% CI 20.9%–27.9%). In an exploratory analysis, hs‐cTn < 5 ng/L identified 295 (41.9%) patients with 98.9% sensitivity (93.9%–100.0%), 99.7% NPV (97.7%–100.0%), and 47.8% specificity (95% CI 43.8%–51.8%).

**Conclusions:**

This novel POC hs‐cTn assay achieves high sensitivity and NPV when used alongside the T‐MACS decision aid, but efficiency may be greater at a 5 ng/L threshold without requiring any decision aid.

**Trial Registration:**

ClinicalTrials.gov identifier: NCT03561051

**Abbreviations**
AMI: acute myocardial infarction
AUC: area under the receiver operating characteristic curve
cTn: cardiac troponin
ECG: electrocardiogram
ED: Emergency Department
EMS: Emergency Medical Services
hs‐cTn: high sensitivity cardiac troponin
MACE: major adverse cardiac events
POC: point of care

## 1 Introduction

Over the past decade there have been significant advances in the approach to diagnosis of acute myocardial infarction (AMI). The development of high‐sensitivity troponin (hs‐cTn) assays, which can detect smaller concentrations of cardiac troponin with greater precision than previous generations of troponin assay, has enabled AMI to be considered “ruled out” for a significant proportion of symptomatic patients (generally between 25% and 45%) on the basis of a single blood test on arrival to the Emergency Department (ED). Strategies by which this can be achieved are principally two‐fold: either employing an unconventionally low “rule‐out” threshold for hs‐cTn, typically at or near the assay limit of detection (LoD) or limit of quantification (LoQ) [1, 2]; or incorporating the hs‐cTn test within a validated decision aid such as the History, Electrocardiogram (ECG), Age, Risk factors and Troponin (HEART) score [3] and the T‐MACS decision aid [4].

While such accelerated diagnostic pathways can provide patients with early reassurance of safety and help unburden crowded EDs, they all require measurement of hs‐cTn in a central laboratory, necessarily requiring patients to attend hospital for diagnosis. This approach still has important limitations and inefficiencies, particularly for patients accessing care via Emergency Medical Services (EMS). Since paramedics do not have access to in vitro diagnostics, all patients with suspected non‐ST elevation myocardial infarction (NSTEMI) are routinely transported to hospital. If paramedics had access to point of care (POC) cardiac troponin testing, very low risk patients could potentially safely avoid hospital conveyance, while high risk patients may be taken directly to specialist cardiac centers.

Until recently, POC troponin assays have lacked the analytical sensitivity and precision of laboratory hs‐cTn assays, limiting their use for AMI rule‐out. Despite this, the primary analysis of the PRESTO study showed that a contemporary POC troponin assay (Roche cobas h232, which is not a high‐sensitivity troponin assay), used with the T‐MACS decision aid, could achieve a negative predictive value (NPV) of 99.1% in the prehospital environment for the determination of a type 1 AMI [5]. However, the formula used to calculate the output of T‐MACS can only classify patients as “very low risk” if the initial troponin concentration is < 10 ng/L. Therefore, our findings relied on an assumption that troponin concentrations < 0.04 ng/mL (equivalent to 40 ng/L, which is the LoD of the assay) were, in fact, less than 10 ng/L. Since then, several new POC troponin assays have become available that achieve the analytical performance criteria to be considered “high‐sensitivity”. In this analysis conducted in the prehospital setting, we evaluate the diagnostic accuracy of a novel POC hs‐cTn assay manufactured by Abbott Point of Care for the determination of index presentation type 1 AMI.

## 2 Methods

### 2.1 Study Design and Setting

This is a pre‐specified secondary analysis from the PRESTO study, a prospective diagnostic test accuracy study conducted in four ambulance NHS Trusts in the United Kingdom, conveying patients to the EDs of 12 hospitals. The PRESTO study protocol has been reported in full detail in the literature [6], as has the primary analysis [5]. PRESTO was approved by the Research Ethics Committee (reference 18/ES/0101).

### 2.2 Participants

All participating paramedics were provided with bespoke training on the study protocol, Good Clinical Practice, venipuncture, and ECG interpretation. Ambulances were then equipped with the necessary consumables to enroll participants in the study in real time.

We included adults (aged ≥ 18 years) who received an EMS response for a primary complaint of pain or discomfort in the chest, epigastrium, jaw, arm(s), neck or throat. Paramedics were able to approach patients for study enrolment if they suspected a diagnosis of AMI. Adults with ECG evidence of ST elevation myocardial infarction (STEMI), those with another condition that would require hospital conveyance in the opinion of the treating paramedic, those who did not have mental capacity to provide informed verbal consent, and those who had experienced no symptoms for more than 24 h prior to paramedic attendance were excluded.

After obtaining verbal consent, paramedics drew 4.5 mL of venous blood into a lithium heparin Vacutainer vial (Becton‐Dickinson, Franklin Lakes, NJ) from an intravenous cannula. After hospital conveyance, and once their condition was sufficiently stable, all participants were asked to provide full written informed consent. If that was not possible prior to leaving hospital, written consent was obtained by witnessed telephone call or by mail.

### 2.3 Data Processing and Collection

#### 2.3.1 Prehospital Environment

Prior to hospital conveyance, paramedics prospectively completed a case report form that included all the data to calculate relevant decision aids. All participants were conveyed to hospital in accordance with standard care guidelines, and the outputs of decision aids and POC troponin testing were not used to influence patient care during the study.

#### 2.3.2 Laboratory Analyses

Paramedics transferred lithium heparin blood samples to ED research teams upon arrival who prepared aliquots of plasma that were frozen within 8 h of sample collection, at −20°C or below for up to 21 days and at −80°C thereafter. Samples were subsequently thawed for analysis in batches by a clinical scientist at the University of Manchester using the Abbott i‐STAT Alinity device and the i‐STAT hs‐TnI cartridge in accordance with the manufacturer's instructions for use. This assay has a 99th percentile upper reference limit (URL) of 13 ng/L in women, 28 ng/L in men, and 21 ng/L overall. In plasma, the coefficient of variation is < 10% at a concentration of 3.70 ng/L. The LoD of the assay in plasma, as determined by the manufacturer, was 1.05 ng/L. The LoQ, defined as the lowest concentration to achieve a coefficient of variation < 20%, was 1.18 ng/L. In accordance with recommendations from the International Federation for Clinical Chemistry Committee for Clinical Applications of Cardiac Biomarkers (IFCC‐CCB) [7], we used sex‐specific 99th percentile URLs and reported hs‐cTnI concentrations in integers using ng/L.

#### 2.3.3 Decision Aids

The outcome of the T‐MACS decision aid and the HEART score were calculated using data recorded contemporaneously by treating paramedics. For T‐MACS, the ECG was recorded as “normal” or “abnormal” (rather than asking if the ECG showed “acute ischaemic changes”), following advice from senior paramedics prior to the start of the study. Both scores were calculated using previously published methods [3, 8], which are fully set out in the statistical analysis plan (SAP, Appendix S1). In brief, we set out to evaluate the HEART score as originally described (troponin component assigned a score of 0 if < URL, 1 if 1–3 × the URL and 2 if > 3 × the URL). We have labeled this as the “original HEART score” as we use the original scoring system, albeit with a high‐sensitivity troponin assay [3], and in a modified version (troponin component scores 0 if < LoD, 1 if between the LoD and the URL and 2 if > URL). This “modified HEART score” has been derived because clinicians may be uncomfortable assigning only one point to patients with a troponin concentration up to three times the URL. It has been reported to have higher sensitivity than the original HEART score [9]. T‐MACS handles hs‐cTn concentration as a continuous variable in the equation to calculate the probability of AMI, and therefore does not have a specific cutoff. However, it can be noted that a patient would only be assigned to the “very low risk” group if the hs‐cTnI concentration is < 10 ng/L, in the absence of any other risk factors.

To calculate metrics of diagnostic test accuracy for decision aids, their outputs were dichotomised as follows: for T‐MACS, we compared the “very low risk” (rule‐out) group (calculated probability < 2%) to all other groups; and for the HEART score (both modified and original) we compared the “low risk” (rule‐out) group (score < 4 points) to all other groups.

The prevalence of type 1 AMI was also stratified by T‐MACS and HEART risk group. T‐MACS risk groups were defined according to the calculated probability: < 2%, very low risk; 2%–4.99%, low risk; 5%–94.99%, moderate risk [3]; 95%, high risk. HEART score risk groups were defined as: 0–3 points, low risk; 4–6 points, moderate risk [3]; 7 points, high risk.

#### 2.3.4 Follow Up

Patients were followed up after 30 days by contacting the participant and/or their general practitioner, and by reviewing all available hospital records.

### 2.4 Outcome Measures

The target condition was an index presentation diagnosis of type 1 AMI, adjudicated by two investigators acting independently, and blinded to all decision aid outcomes and POC troponin results, with reference to a third investigator in the event of disagreements. Adjudicators referred to the fourth universal definition of AMI [10], and classified patients as having type 1 AMI, type 2 AMI, other myocardial injury, or no myocardial injury after review of all relevant clinical data collected in hospital at both the index visit and at 30‐day follow‐up. Primary care data, obtained at 30‐day follow‐up, were also made available to the adjudicators.

Prior to selection, all participating sites were required to confirm that they practised in accordance with national (National Institute for Health and Care Excellence) and/or international (European Society of Cardiology) [11, 12] guidance. Guidance on acceptable criteria for the definition of myocardial injury using the reference standard investigations (the laboratory hs‐cTn test results, measured routinely after hospital conveyance in the respective receiving institutions) was agreed prior to commencing adjudication, and in line with contemporary national and international guidelines [11, 12]. Participants who had incomplete reference standard data according to those criteria were excluded from the analysis.

Secondary outcomes included the occurrence of a type 2 AMI or a major adverse cardiac event (MACE) (a composite of any AMI, death, or coronary revascularization) within 30 days of the initial ambulance call.

### 2.5 Sample Size

Our a priori sample size calculation for the PRESTO study overall was informed by the following parameters, data and assumptions. Based on previous reports, we assumed that a specificity of 45% could be achieved with the index diagnostic test and that the prevalence of type 1 AMI would be approximately 10% [13]. Assuming that we could identify a pathway with 100% sensitivity and 95% confidence intervals (95% CI) with lower bounds above 90% for sensitivity and 99% for NPV, we needed a sample size of 605 participants. Allowing for attrition, we planned to enroll 700 participants.

### 2.6 Statistical Analysis

We set out the principles of statistical analysis and the primary analyses in a master statistical analysis plan (SAP) prior to beginning any examination of data from the PRESTO study. The master SAP stated a priori that sub‐SAPs would subsequently be written and agreed by the chief investigator and the study statistician to guide pre‐specified secondary analyses (including this study) before commencement of said endeavors. Full details of all statistical analyses, including the methods used to calculate outputs of the relevant decision aids, are presented in the sub‐SAP (Appendix S1), and any analyses that were not pre‐stated in the sub‐SAP have therefore been marked as post hoc.

In summary, we analyzed the accuracy of the following care pathways: POC hs‐cTn alone using thresholds set at the 99th percentile sex‐specific URL and the LoD; POC hs‐cTn at the same thresholds plus the paramedic prehospital ECG interpretation; T‐MACS using POC hs‐cTn concentrations; and the HEART score (in both original and modified forms) using POC hs‐cTn concentrations. In addition, we performed a post hoc analysis to determine the maximum hs‐cTnI concentration that could achieve a sensitivity > 98% with > 99% NPV.

We constructed 2 × 2 tables for each of these pathways and calculated sensitivity, specificity, positive predictive value (PPV), and NPV with 95% CI using the Clopper‐Pearson method for sensitivity and specificity and the method described by Mercaldo et al. [14] for predictive values. We calculated 95% CI for likelihood ratios using the “log method” [15]. Pre‐planned subgroup analyses were undertaken stratified by age (< 65 years vs. ≥ 65 years), recorded gender, and time from symptom onset (< 3 h vs. ≥ 3 h).

### 2.7 Patient and Public Involvement

PRESTO included two public members as co‐applicants, co‐investigators and members of the Trial Steering Committee. Both are co‐authors of this manuscript and assisted with all aspects of study design and conduct, and with interpretation and communication of the findings. Two patient groups (the Withington Heart Help Group and the Ticker Club) provided valuable additional input into the design and conduct of the study.

## 3 Results

### 3.1 Characteristics of Study Subjects

**FIGURE 1:**
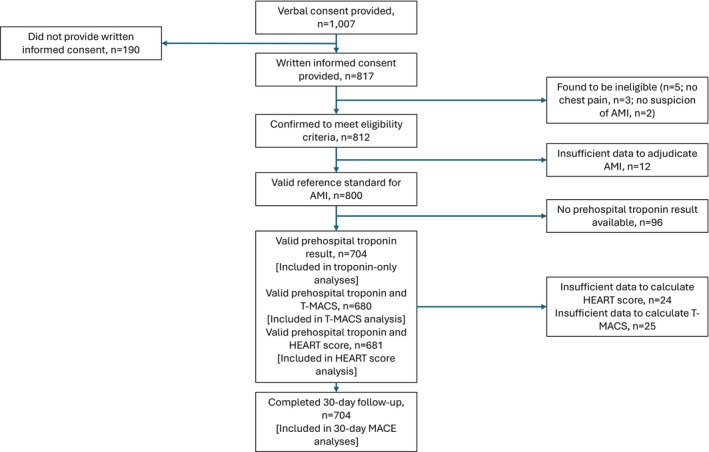
Participant flow diagram.

Between February 2019 and March 2020, a total of 1007 patients provided verbal consent to participate in the PRESTO study, of which 817 subsequently provided written informed consent. After application of pre‐specified exclusion criteria and removal of participants without a valid reference standard hospital laboratory troponin test or a valid result for the investigational hs‐cTn assay (e.g., insufficient sample or sample not processed in time), a total of 704 participants were eligible for analysis (Figure 1), whose baseline characteristics are described in Table 1.

**TABLE 1:** Baseline characteristics of included patients (eligible for primary analysis).

	Total (*n* = 704)	Type 1 AMI (*n* = 89)	No type 1 AMI (*n* = 615)
Age in years, mean (SD)	64.1 (15.1)	68.7 (13.1)	63.4 (15.2)
Recorded gender is female (%)	305 (43.3)	30 (33.7)	275 (44.7)
Recorded gender is male (%)	399 (56.7)	59 (66.3)	340 (55.3)
Previous hyperlipidaemia (%)	165 (23.4)	22 (24.7)	143 (23.3)
Previous hypertension (%)	382 (54.3)	59 (66.3)	323 (52.5)
Previous diabetes (%)	138 (19.6)	25 (28.1)	113 (18.4)
Previous CVA or TIA (%)	71 (10.1)	13 (14.6)	58 (9.4)
Previous peripheral vascular disease (%)	29 (4.1)	7 (7.9)	22 (3.6)
Prior myocardial infarction (%)	209 (29.7)	42 (47.2)	167 (27.2)
Prior PCI or CABG (%)	169 (24.0)	35 (39.3)	134 (21.8)
Family history of heart disease (%)	354 (50.3)	46 (51.7)	308 (50.1)
Current smoking (%)	166 (23.6)	28 (31.5)	138 (22.4)
Time from symptom onset, *n* (%)			
3 h	391 (55.5)	60 (67.4)	331 (53.8)
3–6 h	110 (15.6)	11 (12.4)	99 (16.1)
6 h	184 (26.1)	15 (16.9)	90 (14.9)

The test characteristics of the POC hs‐cTn assay are reported in Table 2. At progressively lower hs‐cTn thresholds, sensitivity increased at the expense of diagnostic efficiency. At our pre‐specified single test rule‐out threshold (< 2 ng/L, LoD), the test had 100.0% sensitivity (95% CI 95.9%–100.0%) but identified only 4% of patients as low risk for type 1 AMI. Therefore, we proceeded to explore the optimal cutoff. This was determined to be < 5 ng/L (sensitivity 98.9%, 95% CI 93.9%–98.9%). As this threshold was not stated a priori in our statistical analysis plan, this was a post hoc analysis.

**TABLE 2:** Test characteristics for AMI.

Index test	Sensitivity (95% CI)	Specificity (95% CI)	PPV (95% CI)	NPV (95% CI)	Number (%) ruled out	Incidence of any 30‐day MACE in patients who would be “ruled out”, *n* (%)
Cardiac troponin (99th percentile cut‐off)	80.9 (71.2–88.5)	88.9 (86.2–91.3)	51.4 (45.3–57.5)	97.0 (95.5–98.0)	564 (80.1)	19/564 (3.4%)
Cardiac troponin (limit of detection cut‐off)	100.0 (95.9–100.0)	4.6 (3.1–6.5)	13.2 (13.0–13.4)	100.0 (87.7–100.0)	28 (4.0)	1/28 (3.6%)
Cardiac troponin (3 ng/L cut‐off)	100.0 (95.9–100.0)	16.6 (13.7–19.8)	14.8 (14.4–15.2)	100.0 (96.5–100.0)	102 (14.5)	2/102 (2.0%)
Cardiac troponin (5 ng/L cut‐off)	98.9 (93.9–100.0)	47.8 (43.8–51.8)	21.5 (20.2–22.9)	99.7 (97.7–100.0)	295 (41.9)	5/295 (1.7%)
Cardiac troponin (99th percentile cut‐off) and ECG [rule out if both normal]	91.0 (83.1–96.0)	54.4 (50.4–58.4)	22.5 (20.7–24.5)	97.7 (95.5–98.8)	333 (47.5)	10/561 (1.8%)
Cardiac troponin (5 ng/L cut‐off) and ECG [rule out if both normal]	98.9 (93.9–100.0)	33.7 (29.9–37.6)	17.8 (16.9–18.7)	99.5 (96.7–99.9)	207 (29.5)	5/294 (1.7%)
T‐MACS	98.9 (94.9–100.0)	22.5 (19.2–26.1)	16.1 (15.5–16.8)	99.3 (95.0–99.9)	134 (19.7)	2/134 (1.5%)
HEART score [original]	92.1 (84.3–96.7)	42.7 (38.6–46.8)	19.2 (17.8–20.7)	97.3 (94.6–98.7)	260 (38.2)	6/260 (2.3%)
HEART score [modified]	93.2 (85.8–97.5)	24.3 (20.9–27.9)	15.4 (14.5–16.4)	96.0 (91.6–98.1)	150 (22.0)	6/150 (4.0%)

Table 2 also demonstrates the test characteristics of POC hs‐cTnI when incorporating into the T‐MACS decision aid, the modified HEART score and the original HEART score. Interestingly, an original HEART score < 4 identified many more patients as low risk than the modified HEART score, 260 (38.2%), but with comparable sensitivity (92.1%, 95% CI 84.3%–96.7%) and NPV (97.3%, 95% CI 94.6%–98.7%).

The incidence of MACE at 30 days in the patients who would have been “ruled out” using each diagnostic pathway evaluated is also reported in Table 2. Of the 295 patients with a prehospital hs‐cTnI < 5 ng/L, five progressed to a MACE at 30 days and there were no deaths. One patient suffered an AMI with cardiac arrest followed by percutaneous coronary intervention (PCI) and four others underwent coronary revascularisation. Of the 134 patients in the T‐MACS “very low risk” group, two underwent PCI but there were no deaths or incident AMIs within 30 days. In the modified HEART score “low risk” group, six of 150 patients underwent PCI but there were no deaths or incident AMIs.

The proportion of patients with type 1 AMI in each T‐MACS and HEART score risk group is shown in Table 3. An important finding to highlight from this analysis is the prevalence of type 1 AMI in the “high risk” groups: with T‐MACS, 83.6% of patients identified as “high risk” had type 1 AMI, versus 34.1% in the corresponding “high risk” group for the HEART (≥ 7) score.

**TABLE 3:** T‐MACS and HEART scores: Number of patients with type 1 AMI and the total number of patients in each risk group.

**T‐MACS**	Very Low risk	Low risk	Moderate risk	High risk
	1/134 (0.7%)	4/70 (5.7%)	38/421 (9.0%)	46/55 (83.6%)

**HEART score (modified)**	Low risk	Moderate risk	High risk
	6/150 (4.0%)	25/364 (6.9%)	57/167 (34.1)

Finally, the diagnostic accuracy of each pathway studied for type 1 or type 2 AMI is shown in the Appendix S1. There were no notable differences in diagnostic accuracy for any of the pathways studied after taking account of type 2 AMIs.

### 3.2 Subgroup Analyses

The full results of subgroup analyses are shown in the Appendix S1. Other than when using the POC hs‐cTnI test at the sex‐specific 99th percentile URL cut‐off, the specificity of each pathway studied was slightly higher in men than in women, in keeping with the slightly higher baseline cardiac troponin concentrations in men. Sensitivity was higher in women than in men when using the sex‐specific 99th percentile URL cut‐off (83.3% vs. 79.7%) and this was also demonstrated in both the original and modified HEART scores (e.g., the modified HEART score < 4 had 100.0% sensitivity in women vs. 89.7% in men). There were no other notable differences in sensitivity between pathways in these subgroup analyses.

Similarly, the subgroup analyses stratified by age showed that specificity was lower in participants aged ≥ 65 years, which is again in keeping with the expected higher baseline troponin concentrations in older adults. A modified HEART score < 4 demonstrated a lower sensitivity (80%) in adults < 65 years compared with 100% in participants aged ≥ 65 years.

Among participants assessed within 3 h of symptom onset, the sensitivity of hs‐cTnI alone, and of T‐MACS was similar to that in the overall group. The sensitivity of both the original and modified HEART scores was 91.7% (Table S6).

## 4 Discussion

In patients presenting to paramedics in the prehospital setting with chest pain suspicious for an AMI, we present some of the first evidence describing the potential benefit of incorporating a novel commercially available POC hs‐cTnI assay into the assessment and management of these patients in this environment. Using the Abbott Next generation POC hs‐cTnI assay alone, at thresholds close to the LOD or incorporated into validated risk‐assessment tools such as T‐MACS, it seems feasible to identify significant proportions of patients, perhaps up to 4 in 10, as having AMI “ruled out” with high sensitivity and NPV.

The portability of the i‐Stat Alinity device (Abbott Point of Care) coupled with the relatively short turnaround time of the Abbott Next generation POC hs‐cTnI assay (approximately 15 min) and previous evidence of successful device use for other conditions in the prehospital setting [16], makes it attractive for use in this challenging environment.

In the SAP, we set out a number of a priori analyses. These included hs‐cTnI cut‐offs set at the 99th percentile sex‐specific URL, the assay LoD, and previously validated decision aids (T‐MACS and the HEART score). Of these, the LoD achieved high sensitivity but very low specificity, identifying very few patients as having AMI considered “ruled out”, limiting the value of such an approach in practice. As we may have anticipated, the 99th percentile URL provided insufficient sensitivity to permit single sample “rule out”. Further, a low‐risk HEART score, even when modified to yoke the benefits of hs‐cTn tests, could only achieve 93.1% sensitivity and 96.0% NPV for type 1 AMI. A 4% post‐test probability of type 1 AMI for the “low risk” HEART score group means it is unlikely that this pathway could be safely implemented in practice. The “very low risk” outcome of the T‐MACS decision aid identified about 1 in 5 patients as perhaps being suitable for non‐conveyance to hospital, with 98.9% sensitivity and 99.3% NPV for a type 1 AMI. This finding is similar to the conclusion of the primary analysis from the PRESTO study, in which we utilized a contemporary POC cardiac troponin assay [5]. In that analysis, the limited analytical sensitivity of the POC assay evaluated required us to assume that all cardiac troponin concentrations < 0.04 ng/mL (< 40 ng/L) were in fact < 0.01 ng/mL (< 10 ng/L) to identify any patients as being “very low risk” with the T‐MACS decision aid. The high‐sensitivity assay evaluated here does not require such an assumption and would therefore be preferable for future clinical use.

Additionally, in our SAP, we also stated the goal to derive the optimal hs‐cTnI cut‐off threshold for single sample rule‐out with this POC hs‐cTnI assay, aiming to achieve sensitivity > 98% and NPV > 99%. At a cut‐off of < 5 ng/L, the assay identified about twice as many patients as the T‐MACS “very low risk” outcome, about 2 in 5, for potential non‐conveyance to hospital, but with very similar, and still excellent, diagnostic performance (98.9% sensitivity with 99.7% NPV). While this analysis must be considered exploratory (because we derived this cut‐off within the same cohort), our results suggest that the POC hs‐cTnI assay alone may have advantages over any decision aid evaluated, and may be the optimal pathway to “rule out” AMI with a single test in this environment. One study, conducted in the ED, also found that using hs‐cTn alone had greater diagnostic efficiency than the HEART score, without sacrificing sensitivity [17]. T‐MACS, however, has previously been shown to have greater diagnostic efficiency than hs‐cTn alone [4, 8]. Our finding that this POC hs‐cTnI assay alone had greater diagnostic efficiency than T‐MACS is therefore particularly interesting. The simplicity of that approach may be attractive for practicing paramedics operating in a high‐pressure environment.

### 4.1 Strengths and Limitations

Strengths of this evaluation include its prospective, multi‐center design; the real‐time data collection by paramedics; inclusion based on paramedic suspicion of AMI, reflecting real world practice; and the clinical relevance of our primary outcome measure, addressing the main question faced by paramedics in this setting. We also accounted for important secondary safety measures.

One limitation of our evaluation is that we drew blood samples in the prehospital environment and transported them to hospital, where plasma was separated and frozen pending subsequent analysis. While this permitted us to undertake a multi‐center diagnostic test accuracy study in a timely manner, it did mean that the POC hs‐cTnI assay was evaluated in plasma, rather than whole blood. While the differences in measurements between the two sample types are likely to be minor, our findings do require prospective validation in whole blood samples. Further, it will be important to evaluate the performance of the device when tests are run by paramedics in the prehospital environment, and to explore the perspectives of paramedics and patients on proposed changes to the care pathway.

Further, while the 5 ng/L hs‐cTnI cut‐off appears to be optimal based on our findings, it is important to recognize that it was derived in this cohort, raising the possibility of overfitting the results of this analysis, and again highlighting the importance of prospective validation prior to clinical implementation.

Finally, it is important to recognize the width of the 95% confidence intervals, particularly around sensitivity. In a survey of emergency physicians, the majority of respondents would have accepted a 0.5% or less risk of missed AMI or 30‐day MACE [18]. While assays that are currently used in practice do not achieve this, it emphasizes the need for further prospective research to evaluate this novel POC hs‐cTn assay. Further on this point, it is important to acknowledge that the study was not powered to directly compare the sensitivity of the different pathways, and therefore we cannot definitively conclude that the sensitivity of any of the three pathways evaluated is superior.

### 4.2 Future Directions

In addition to prospective validation using whole blood samples, it will be important to evaluate the practical usability of this POC test from the perspective of paramedics, and to evaluate the clinical effectiveness of the pathways evaluated when the devices are used in the field. Successful implementation of pathways to facilitate non‐conveyance will relieve pressure on EMS and crowded EDs, improving response times and eliminating unnecessary waiting and anxiety for patients. Meanwhile, patients who are identified as being at “high risk” of AMI may be transported directly to specialist cardiology centers, expediting definitive care and reducing unnecessary secondary ambulance transfers.

## 5 Conclusions

The Abbott i‐Stat hs‐cTnI assay has high sensitivity and NPV for AMI in the prehospital environment when used as part of the T‐MACS decision aid and using a cut‐off of 5 ng/L. If our findings can be prospectively validated using whole blood samples, our findings suggest that a single hs‐cTnI measurement < 5 ng/L could highlight approximately 40% of patients as being potentially eligible for safe non‐conveyance to hospital. Future work should focus on prospective validation of these pathways using whole blood samples, analysis of the usability of the assay from the perspective of paramedics, and evaluation of clinical and cost effectiveness.

## Author Contributions

R.B. and J.G.C. drafted the manuscript. A.A., E.C., E.C., A.N.S., J.P., and R.B. designed this study. As trial statistician, M.H. prepared the database for analysis and agreed both the master statistical analysis plan and the sub‐statistical analysis plan. Statistical analysis was then undertaken by R.B. The following authors lead PRESTO sites: S.B. (NWAS), J.G.C. (Aberdeen), K.K. (SWAS), and A.R. (WMAS). All authors contributed, read, and approved the final manuscript and agree to be accountable for all aspects of the work.

## Funding

The study was funded by the National Institute for Health and Care Research, Research for Patient Benefit scheme (reference PB‐PG‐1216‐20034). This analysis was also supported by an investigator‐initiated research grant from Abbott Point of Care.

## Disclosure

No large language model has been used in the preparation of this manuscript.

## Supporting information


**Appendix S1:** acem70213‐sup‐0001‐AppendixS1.docx.
**Table S1:** Local laboratory troponin assays by site.
**Table S2:** Test characteristics for a diagnosis of type 1 or type 2 AMI.
**Table S3:** Diagnostic accuracy for type 1 AMI in patients with recorded gender of female (all participants had a recorded gender of either male or female, with no participants declaring nonbinary or transgender status).
**Table S4:** Diagnostic accuracy for type 1 AMI in patients with recorded gender of male (all participants had a recorded gender of either male or female, with no participants declaring nonbinary or transgender status).
**Table S5:** Diagnostic accuracy for type 1 AMI in patients aged at least 65 years.
**Table S6:** Diagnostic accuracy for type 1 AMI in patients aged less than 65 years.
**Table S7:** Diagnostic accuracy for type 1 AMI in patients with time from symptom onset < 3 hours.

## Data Availability

The data that support the findings of this study are available on request from the corresponding author. The data are not publicly available due to privacy or ethical restrictions.
