# Supplementary material for: Diagnostic Accuracy of a Novel Point of Care High‐Sensitivity Troponin Assay in the Prehospital Environment
Source: Acad Emerg Med. 2026 Jan 13;33(1):e70213. doi: 10.1111/acem.70213 (PMC12798273; doi:10.1111/acem.70213)
Supplement: Supplementary file 1 — Appendix S1: acem70213‐sup‐0001‐AppendixS1.docx. Table S1: Local laboratory troponin assays by site. Table S2: Test characteristics for a diagnosis of type 1 or type 2 AMI. Table S3: Diagnostic accuracy for type 1 AMI in patients with recorded gender of female (all participants had a recorded gender of either male or female, with no participants declaring nonbinary or transgender status). Table S4: Diagnostic accuracy for type 1 AMI in patients with recorded gender of male (all participants had a recorded gender of either male or female, with no participants declaring nonbinary or transgender status). Table S5: Diagnostic accuracy for type 1 AMI in patients aged at least 65 years. Table S6: Diagnostic accuracy for type 1 AMI in patients aged less than 65 years. Table S7: Diagnostic accuracy for type 1 AMI in patients with time from symptom onset < 3 hours. [file ACEM-33-0-s001.docx]

Table 1: Local laboratory troponin assays by site

| Site # | Trust/Site | Assay |
| --- | --- | --- |
| 01 | Manchester University NHS Foundation Trust | Roche hs-cTnT |
| 02 | Salford Royal NHS Foundation Trust | Siemens TNIH |
| 03 | Bolton NHS Foundation Trust | Roche hs-cTnT |
| 05 | North Bristol NHS Trust | Roche hs-cTnT |
| 06 | Royal Devon and Exeter NHS Foundation Trust | Roche hs-cTnT |
| 07 | University Hospitals Plymouth NHS Trust | Abbott hs-cTnI |
| 08 | Taunton and Somerset NHS Foundation Trust | Roche hs-cTnT |
| 10 | University Hospitals Coventry and Warwickshire NHS Trust | Roche hs-cTnT |
| 11 | Wigan | Siemens TNIH |
| 12 | Warwick | Roche hs-cTnT |
| 13 | Aberdeen Royal Infirmary | Siemens troponin I Ultra |

# Secondary analyses

Table 2: Test characteristics for a diagnosis of type 1 or type 2 AMI

| **Index test** | **Sensitivity (95% CI)** | **Specificity**  **(95% CI)** | **PPV**  **(95% CI)** | **NPV**  **(95% CI)** |
| --- | --- | --- | --- | --- |
| Cardiac troponin (99^th^ percentile cut-off) | 81.6  (72.7 – 88.5) | 90.7  (88.1 – 92.9) | 60.0  (53.5 – 66.2) | 96.6  (95.0 – 97.7) |
| Cardiac troponin (limit of detection cut-off) | 100.0  (96.5 – 100.0) | 4.7  (3.1 – 6.7) | 15.2  (15.0 -15.5) | 100.0  (87.7 – 100.0) |
| Cardiac troponin (5ng/L cut-off) | 99.0  (94.7 – 100.0) | 48.9  (44.9 – 53.0) | 24.9  (23.5 – 26.5) | 99.7  (97.7 – 100.0) |
| T-MACS | 99.0  (94.7 – 100.0) | 23.1  (19.7 – 26.7) | 18.7  (18.0 – 19.4) | 99.3  (95.0 – 99.9) |
| HEART score [original] | 93.0  (86.1 – 97.1) | 43.6  (39.5 – 47.7) | 22.1  (20.6 – 23.7) | 97.3  (94.6 – 98.7) |
| HEART score [modified] | 94.0  (87.4 – 97.8) | 24.8  (21.3 – 28.5) | 17.7  (16.7 – 18.7) | 96.0  (91.6 – 98.1) |

# Subgroup analyses

## Subgroup analyses stratified by recorded gender

Table 3: Diagnostic accuracy for type 1 AMI in patients with recorded gender of female (all participants had a recorded gender of either male or female, with no participants declaring nonbinary or transgender status)

| **Index test** | **Sensitivity (95% CI)** | **Specificity**  **(95% CI)** | **PPV**  **(95% CI)** | **NPV**  **(95% CI)** | **Number (%) ruled out** | **Incidence of any 30-day MACE in patients who would be ‘ruled out’, n (%)** |
| --- | --- | --- | --- | --- | --- | --- |
| Cardiac troponin (99^th^ percentile cut-off) | 83.3  (65.3 – 94.4) | 89.1  (84.8 – 92.5) | 45.5  (36.5 – 54.8) | 98.0  (95.7 – 99.1) | 250  (82.0) | 6 / 250  (2.4%) |
| Cardiac troponin (limit of detection cut-off) | 100.0  (88.4 – 100.0) | 8.0  (5.1 – 11.9) | 10.6  (10.3 – 10.9) | 100.0  (84.6 – 100.0) | 22  (7.2) | 1 / 22  (4.5%) |
| Cardiac troponin (5ng/L cut-off) | 100.0  (88.4 – 100.0) | 55.6  (49.6 – 61.6) | 19.7  (17.7 – 21.9) | 100.0  (97.6 – 100.0) | 153  (50.2) | 1 / 153  (0.7%) |
| T-MACS | 96.7  (82.8 – 99.9) | 27.6  (22.3 – 33.4) | 13.1  (12.0 – 14.3) | 98.7  (91.3 – 99.8) | 74  (25.1) | 2 / 74  (2.7%) |
| HEART score [original] | 96.7  (82.8 – 99.9) | 47.9  (41.8 – 54.1) | 17.3  (15.5 – 19.2) | 99.2  (94.9 – 99.9) | 129  (43.4) | 0 /129  (0.0%) |
| HEART score [modified] | 100.0  (88.4 – 100.0) | 28.8  (23.5 – 34.7) | 13.6  (12.8 – 14.6) | 100.0  (95.3 – 100.0) | 77  (25.9) | 0 / 77  (0.0%) |

Table 4: Diagnostic accuracy for type 1 AMI in patients with recorded gender of male (all participants had a recorded gender of either male or female, with no participants declaring nonbinary or transgender status)

| **Index test** | **Sensitivity (95% CI)** | **Specificity**  **(95% CI)** | **PPV**  **(95% CI)** | **NPV**  **(95% CI)** | **Number (%) ruled out** | **Incidence of any 30-day MACE in patients who would be ‘ruled out’, n (%)** |
| --- | --- | --- | --- | --- | --- | --- |
| Cardiac troponin (99^th^ percentile cut-off) | 79.7  (67.2 – 89.0) | 88.8  (85.0 – 92.0) | 55.3  (47.2 – 63.2) | 96.2  (93.8 – 97.7) | 314  (78.7) | 13 / 314  (4.1%) |
| Cardiac troponin (limit of detection cut-off) | 100.0  (93.9 – 100.0) | 8.0  (5.1 – 11.9) | 10.6  (10.3 – 10.9) | 100.0  ( – 100.0) | 6  (1.5) | 0 / 6  (0.0%) |
| Cardiac troponin (5ng/L cut-off) | 98.3  (90.9 – 100.0) | 41.5  (36.2 – 46.9) | 22.6  (20.9 – 24.3) | 99.3  (95.3 – 99.9) | 142  (35.6) | 4 / 142  (2.8%) |
| T-MACS | 100.0  (93.9 – 100.0) | 18.4  (14.4 – 23.1) | 18.2  (17.4 – 18.9) | 100.0  (94.0 – 100.0) | 60  (15.6) | 0 / 60  (0.0%) |
| HEART score [original] | 89.7  (78.8 – 96.1) | 38.3  (33.0 – 43.9) | 20.6  (18.6 – 22.6) | 95.4  (90.6 – 97.8) | 131  (34.1) | 6 / 131  (4.6%) |
| HEART score [modified] | 89.7  (78.8 – 96.1) | 20.6  (16.3 – 25.4) | 16.7  (15.3 – 18.2) | 91.8  (83.6 – 96.1) | 73  (19.0) | 6 / 73  (8.2%) |

Table 5: Diagnostic accuracy for type 1 AMI in patients aged at least 65 years

| **Index test** | **Sensitivity (95% CI)** | **Specificity**  **(95% CI)** | **PPV**  **(95% CI)** | **NPV**  **(95% CI)** | **Number (%) ruled out** | **Incidence of any 30-day MACE in patients who would be ‘ruled out’, n (%)** |
| --- | --- | --- | --- | --- | --- | --- |
| Cardiac troponin (99^th^ percentile cut-off) | 79.7  (67.2 – 89.0) | 82.3  (77.5 – 86.4) | 47.0  (40.2 – 53.9) | 95.4  (92.5 – 97.2) | 258 / 358  (72.1%) | 12 / 258  (4.7%) |
| Cardiac troponin (limit of detection cut-off) | 100.0  (93.9 – 100.0) | 0.7  (0.1 – 2.4) | 16.6  (16.4 – 16.7) | 100.0  (15.8 – 100.0) | 2 / 358  (0.6%) | 0 / 2  (0.0%) |
| Cardiac troponin (5ng/L cut-off) | 100.0  (93.9 – 100.0) | 23.4  (18.7 – 28.6) | 20.5  (19.5 – 21.5) | 100.0  (94.9 – 100.0) | 70 / 358  (19.6%) | 1 / 70  (1.4%) |
| T-MACS | 98.3  (90. – 100.0) | 15.6  (11.5 – 20.3) | 19.5  (18.6 – 20.5) | 97.8  (86.1 – 99.7) | 45 / 342  (13.2%) | 1 / 45  (2.2%) |
| HEART score [original] | 100.0  (93.8 – 100.0) | 20.6  (16.1 – 25.8) | 20.4  (19.4 – 21.3) | 100.0  (93.9 – 100.0) | 59 / 344  (17.2%) | 0 / 59  (0.0%) |
| HEART score [modified] | 100.0  (93.8 – 100.0) | 5.9  (3.5 – 9.4) | 17.7  (17.3 – 18.2) | 100.0  (80.5 – 100.0) | 17 / 344  (4.9%) | 0 / 17  (0.0%) |

Table 6: Diagnostic accuracy for type 1 AMI in patients aged less than 65 years

| **Index test** | **Sensitivity (95% CI)** | **Specificity**  **(95% CI)** | **PPV**  **(95% CI)** | **NPV**  **(95% CI)** | **Number (%) ruled out** | **Incidence of any 30-day MACE in patients who would be ‘ruled out’, n (%)** |
| --- | --- | --- | --- | --- | --- | --- |
| Cardiac troponin (99^th^ percentile cut-off) | 83.3  (65.3 – 94.4) | 95.3  (92.3 – 97.3) | 62.5  (49.8 – 73.7) | 98.4  (96.4 – 99.3) | 306 / 346  (88.4%) | 7 / 306  (2.3%) |
| Cardiac troponin (limit of detection cut-off) | 100.0  (88.4 – 100.0) | 8.2  (5.5 – 11.8) | 9.4  (9.1 – 9.7) | 100.0  (86.8 – 100.0) | 26 / 346  (7.5%) | 1 / 26  (3.8%) |
| Cardiac troponin (5ng/L cut-off) | 96.7  (82.8 – 99.9) | 70.9  (65.5 – 75.8) | 24.0  (20.8 – 27.5) | 99.6  (97.0 – 99.9) | 225 / 346  (65.0%) | 4 / 225  (1.8%) |
| T-MACS | 100.0  (88.4 – 100.0) | 28.9  (23.9 – 34.3) | 12.1  (11.3 – 12.8) | 100.0  (95.9 – 100.0) | 89 / 338  (26.3%) | 1 / 89  (1.1%) |
| HEART score [original] | 76.7  (57.7 – 90.1) | 63.2  (57.5 – 68.6) | 16.9  (13.7 – 20.7) | 96.5  (93.5 – 98.2) | 201 / 337  (59.6%) | 6 / 201  (3.0%) |
| HEART score [modified] | 80.0  (61.4 – 92.3) | 41.4  (35.8 – 47.1) | 11.8  (9.8 – 14.0) | 95.5  (91.1 -97.8) | 133 / 337  (39.5%) | 6 / 133  (4.5%) |

Table 7: Diagnostic accuracy for type 1 AMI in patients with time from symptom onset <3 hours

| **Index test** | **Sensitivity (95% CI)** | **Specificity**  **(95% CI)** | **PPV**  **(95% CI)** | **NPV**  **(95% CI)** | **Number (%) ruled out** | **Incidence of any 30-day MACE in patients who would be ‘ruled out’, n (%)** |
| --- | --- | --- | --- | --- | --- | --- |
| Cardiac troponin (99^th^ percentile cut-off) | 75.0  (62.1 – 85.3) | 90.0  (86.3 – 93.0) | 57.7  (48.9 – 66.1) | 95.2  (92.8 – 96.9) | 313 / 391  (80.1%) | 16 / 313  (5.1%) |
| Cardiac troponin (limit of detection cut-off) | 100.0  (94.0 – 100.0) | 4.2  (2.3 – 7.0) | 15.9  (15.6 -16.2) | 100.0  (76.8 – 100.0) | 14 / 391  (3.6%) | 0 / 14  (0/0%) |
| Cardiac troponin (5ng/L cut-off) | 98.3  (91.1 – 100.0) | 46.2  (40.8 – 51.8) | 24.9  (23.0 – 26.9) | 99.4  (95.6 – 99.9) | 154 / 391  (39.4%) | 3 / 154  (1.9%) |
| T-MACS | 98.3  (91.1 – 100.0) | 18.6  (14.5 – 23.3) | 18.4  (17.5 – 19.3) | 98.4  (89.5 – 99.8) | 61 / 382  (16.0) | 1 / 61  (1.6%) |
| HEART score [original] | 91.7  (81.6 -97.2) | 43.5  (38.0 – 49.2) | 23.5  (21.4 – 25.8) | 96.5  (92.2 – 98.5) | 143 / 377  (37.9%) | 5 / 143  (4.1%) |
| HEART score [modified] | 91.7  (81.6 – 97.2) | 24.3  (19.7 – 29.4) | 18.6  (17.2 – 20.2) | 93.9  (86.7 – 97.3) | 82 / 377  (21.8%) | 5 / 82  (6.1%) |
